# Anonymous Diaper-Based Meconium Collection for Ethyl Glucuronide Analysis: A Pilot Feasibility Study

**DOI:** 10.3390/toxics14050413

**Published:** 2026-05-09

**Authors:** Mirjami Jolma, Mikko Koivu-Jolma, Nunzia La Maida, Simona Pichini, Adele Minutillo, Ilona Autti-Rämö, Hanna Kahila

**Affiliations:** 1 Päijät-Häme Central Hospital, University of Helsinki, 00014 Helsinki, Finland; mirjami.jolma@helsinki.fi; 2 Faculty of Science, University of Helsinki, 00014 Helsinki, Finland; mikko.koivu-jolma@helsinki.fi; 3 Department of Environmental and Biological Sciences, University of Eastern Finland, 80100 Joensuu, Finland; 4 National Centre on Addiction and Doping, Istituto Superiore di Sanità, 00161 Rome, Italy; nunzia.lamaida@iss.it (N.L.M.); simona.pichini@iss.it (S.P.); 5 Faculty of Medicine, University of Helsinki, 00014 Helsinki, Finland; ilona.autti@gmail.com; 6 Obstetrics and Gynecology, Helsinki University Hospital, 00029 Helsinki, Finland; hanna.kahila@hus.fi

**Keywords:** prenatal alcohol exposure, ethyl glucuronide, meconium

## Abstract

Prenatal alcohol exposure (PAE) is a major public health concern, yet maternal self-reporting remains unreliable. Meconium accumulates alcohol metabolites during the second half of gestation, and ethyl glucuronide (EtG) is considered a stable and specific biomarker of late-pregnancy alcohol exposure. Diapers containing meconium may serve as a noninvasive method for collecting anonymous biological samples, which could help reduce participation bias in studies of this sensitive subject. This pilot study assessed the feasibility of anonymously collecting meconium-containing diapers for subsequent quantitative EtG analysis after extended periods of frozen storage and international transport, and examined intra-pair consistency in samples from the same newborn. Mothers collected their newborns’ diapers, which were then divided into two aliquots (A and B) by a study assistant and stored at −80 °C until analysis. Out of 178 samples collected from 105 infants, 137 samples were analyzable. Eleven samples exceeded the limit of quantification (10 ng/g), while two samples (one pair) surpassed the 30 ng/g cutoff, indicating significant PAE. EtG concentrations showed high intra-pair agreement, supporting the robustness of the biomarker and analytical method. Anonymous diaper-based meconium collection is feasible but operationally demanding. Although the low participation rate and methodological factors, including sample loss, precluded true prevalence estimation, making it exploratory, the detection of quantifiable EtG in 8% of analyzable samples suggests that PAE remains an issue.

## 1. Introduction

Prenatal alcohol exposure is associated with a broad spectrum of adverse outcomes, including neurodevelopmental, somatic, and behavioral impairments [1], and is the underlying cause of fetal alcohol spectrum disorders (FASD) [2]. Epidemiological estimates indicate that up to one in four pregnancies in Europe may involve alcohol use [3]. However, self-reported alcohol consumption during pregnancy is widely recognized to be substantially underestimated, particularly in contexts where abstinence is recommended. Biomarker-based studies, including maternal hair analysis, have highlighted significant discrepancies between self-report and objective measures of exposure [4].

Meconium, commonly known as the initial fecal matter produced by a newborn, is a dark greenish substance that accumulates in the fetal intestine during the latter stages of pregnancy. It begins to form between approximately the 12th and 26th gestational weeks and, unlike blood-based samples, may reflect continuous exposure to toxicants throughout pregnancy, providing a wider window for detecting long-term in utero exposure. It contains a complex mixture of metabolites and residues reflecting substances to which the fetus has been exposed throughout gestation, including nutrients, potential medications, illicit drugs, alcohol, and other environmental influences. Owing to this unique composition, meconium provides valuable insights into the prenatal environment and the overall health of the fetus. Compared with umbilical cord blood, meconium has been shown to be a more suitable biological matrix for analyzing ethyl glucuronide and other potential toxicants because it integrates exposures over time and contains higher concentrations of metabolites. This is related to its physiological origin as a product of fetal digestion, which may also include bile components; bile represents a major route of metabolite elimination alongside urine [5,6]. Over the past decade, its analysis has become a well-established approach for detecting ethanol biomarkers, facilitating the identification of prenatal alcohol exposure (PAE) [7].

Traditionally, fatty acid ethyl esters (FAEEs) have been utilized for this purpose; however, their clinical reliability may be limited by poor stability [8,9] and vulnerability to confounding variables, such as maternal dietary habits [10]. In contrast, EtG, a direct metabolite of ethanol, demonstrates greater stability and higher diagnostic specificity than FAEEs [11,12]. Since 2015, an EtG cutoff of 30 ng/g has been internationally accepted to distinguish between significant neonatal exposure and non-exposure to maternal alcohol [11].

Despite advances in biomarker detection, the accurate assessment of prenatal exposure remains challenging. Maternal self-reporting of alcohol and other substance use is frequently affected by social desirability bias, recall errors, and concerns about stigma or potential consequences, resulting in systematic underreporting and exposure misclassification [13,14]. These issues not only compromise data accuracy but also reduce voluntary participation in screening programs, as both neonatal testing (e.g., meconium analysis) and maternal assessments (e.g., hair analysis) can be seen as intrusive or judgmental. As a result, participation bias may further limit the representativeness of study populations.

In this context, the use of meconium retrieved from routinely discarded disposable diapers represents a practical and non-invasive alternative for biomonitoring prenatal exposure. As this biological matrix is already available within standard neonatal care, the use of such a matrix eliminates the need for additional sampling procedures and reduce discomfort for both mother and child. Moreover, adopting anonymous testing protocols based on such samples may help to overcome important barriers to participation. By ensuring the removal of personal identifiers and safeguarding confidentiality, this approach can reduce stigma-related concerns and foster greater trust. As a result, anonymity has the potential to enhance participation rates, promote inclusivity, and improve the representativeness of collected data. Ultimately, integrating anonymous, non-invasive biomarker screening into routine clinical practice may strengthen the feasibility and epidemiological validity of public health surveillance in the perinatal context.

This pilot study primarily aimed to evaluate the feasibility and the manageability of a study protocol based on anonymously collecting meconium-containing diapers for EtG analysis and to assess the consistency of EtG measurements in paired samples from the same newborn as an indicator of analytical reliability.

An additional secondary exploratory objective was to estimate the prevalence of quantifiable EtG in neonatal meconium as an indicator of maternal alcohol exposure among participating mother–infant pairs.

## 2. Materials and Methods

### 2.1. Study Setting, Recruitment and Ethics

The study was carried out at the Women’s Hospital of Helsinki University Hospital (HUS). Inclusion criteria for recruitment were as follows: the mother had to be able to understand the written study information and the anonymous consent form in Finnish, the newborn had to stay with the mother in the postpartum ward, and the infant must not have transitioned from meconium to later stool yet.

Infants admitted to the neonatal unit due to prematurity, congenital anomalies, or other medical complications, were excluded.

Participation was voluntary and anonymous. Nurses and midwives informed eligible mothers about the study and provided study materials, including written study information, instructions, sealable opaque bags, a substance-use questionnaire, and anonymous consent forms, which were distributed to postnatal wards. Mothers identified by nursing staff upon arrival at the postpartum ward were asked to read the study information, provide informed consent, and self-report their use of alcohol, nicotine, and psychoactive medication during the latter half of pregnancy by selecting predefined response options. Each mother stayed in a private single room with her infant, allowing her to review the study materials, complete the form, and collect the sample independently. Healthcare staff were available as needed but did not interfere with form completion or sample collection.

### 2.2. Sample Collection and Handling

The hospital supplied all participants with standardized diapers. Consequently, the brand, type, and material were uniform across all samples. Participants placed a diaper containing visible meconium into a sealable plastic bag. The bag and consent form, also sealed, were returned to staff and stored in a designated refrigerator. A study assistant collected the bags and transported them to a nearby laboratory where two aliquots (A and B) were scooped from each diaper and stored in labeled containers. Only the first meconium sample passage after birth was collected from each infant; any subsequent stool passages were excluded. Aliquots A and B were derived from the same diaper specimen and thus constitute technical replicates, rather than independent samples originating from separate bowel movements. Samples and consent forms were assigned matching numeric codes to maintain anonymity while enabling pairing. Samples were stored at −80 °C.

Collection during weekends (between Friday afternoon to Monday afternoon) was not possible. Consequently, samples collected on Mondays could have remained refrigerated in bagged diapers for up to three days prior to scooping and freezing. These samples collected on Mondays were recorded and analyzed as a separate subgroup to assess potential effects of delayed freezing.

### 2.3. Storage and Transport

Due to administrative and logistical challenges, storage time exceeded expectations. No domestic laboratory could provide high-quality quantitative EtG analysis at reasonable cost. After extensive procedures, permits were obtained to transport frozen samples to Italy for analysis in an accredited laboratory experienced in meconium EtG quantification. Samples were transported under controlled frozen conditions.

### 2.4. Laboratory Analysis

Prior to the analytical procedure, sample quality was assessed by a laboratory technician. Sample drying was evaluated during laboratory processing by visual inspection; samples were considered excessively dried when meconium was fully absorbed into the diaper matrix and could not be recovered without contamination by cotton fibers. Samples yielding less than 100 mg of recoverable material were excluded from analysis. EtG was measured using a previously validated GC–MS/MS analytical method with minor modifications, including the adjustment of the limit of quantification to 10 ng/g [15].

Briefly, 100 mg of recovered meconium (diaper-free) were spiked with an internal standard (EtGd5) and acetonitrile, then ultrasonicated at 40 °C. The supernatant was purified using NH_2_ solid-phase extraction cartridges (Agilent Technologies, Santa Clara, CA, USA), eluted with methanolic HCl, and evaporated to dryness under nitrogen. The residue was subsequently derivatized with BSTFA (Merck, Darmstadt, Germany) and injected into a GC-MS/MS system (Agilent Technologies, Santa Clara, CA, USA).

## 3. Results

### 3.1. Recruitment and Participation Rate

In 2021, a total of 9209 infants were born at the hospital. Approximately 20% of births (*n* ≈ 1840) occurred during the data collection period. Of these, an estimated 75% (*n* ≈ 1380) were born to Finnish-speaking mothers. After exclusion of preterm infants and those requiring neonatal unit care for other reasons (estimated at approximately 15% of births within the study period), around 1170 infants met the eligibility criteria. A total of 105 consent forms were obtained, corresponding to an estimated participation rate of approximately 9% (95% CI 7–11%) among eligible mother–infant pairs.

### 3.2. Sample Collection and Analytical Eligibility

In total, 178 diaper-derived samples were collected, subsequently frozen, and transported for analysis. For 52 infants, two samples, identified as paired aliquots (A and B), were available. Three samples shared the same numeric code, suggesting either three subsamples from a single infant or a labeling inconsistency. The remaining samples were submitted as single specimens.

Following long-term frozen storage and international transport, eight samples could not be reliably identified or linked to consent forms due to partial fading or ambiguity of permanent marker labels. These samples were analyzed as unpaired specimens without associated questionnaire data or information on the collection day.

Prior to analysis, 41 samples were excluded due to insufficient quantity or excessive drying, which precluded reliable extraction and measurement (Table 1). Overall, 137 samples were deemed suitable for laboratory analysis.

### 3.3. EtG Analysis

Table 2 shows the results of 137 analyzed individual samples, categorized as single samples, pairs, one out of two from pairs, triplicates, and unidentified. Detailed sample-level data and statistical analyses are provided in Supplementary Data S1.

A substantial majority of the samples, 92% (95% CI 87–97%), displayed EtG concentrations below 10 ng/g. As the cut-off thresholds increased (10, 20, and 30 ng/g), the proportion of positive samples decreased. 6.6% (95% CI 2–11%) of samples fell within the intermediate concentration range of 10.4–19.3 ng/g, while only 1.5% (95% CI 0–3%) exceeded 30 ng/g, indicating likely significant prenatal alcohol exposure. Among the paired samples, one infant showed EtG levels ≥30 ng/g in both aliquots A and B.

Among 81 participants (excluding unidentified samples) with at least one analyzable sample, 7 (8.6%, 95% CI 3–15%) had EtG ≥ LOQ.

#### Intra-Pair Consistency

Of 46 analyzable pairs, 45 (97.8%) had EtG levels concordant. One pair had aliquot B close to the LOQ (10.4 ng/g) and aliquot A below it, which is within the expected 10% method accuracy. Following method modification, the analytical approach demonstrated acceptable intra- and inter-assay precision within OSAC validation criteria [16], supporting the robustness of the measurements.

### 3.4. Self-Report vs. EtG

Among the 105 anonymous consent and self-report forms collected, 90.5% indicated no use of alcohol, nicotine products, or psychoactive drugs during the second half of pregnancy, while 3.8% did not respond to the substance-use questions. Alcohol use, nicotine product use, and psychoactive drug use were each reported by two participants. Overall, 5.7% of participants reported use of at least one substance, with no reports of use across multiple categories.

Of the two participants who reported alcohol use, one showed low but quantifiable EtG concentrations in both paired samples (12.3 and 14.1 ng/g), whereas the other sample was not analyzable. Participants reporting nicotine product or psychoactive drug use had EtG concentrations below the LOQ, except for one participant reporting psychoactive drug use whose sample was not analyzable. Among the four participants who did not respond to the substance-use questions, one had EtG concentrations exceeding 30 ng/g in both paired samples (36.5 and 34.9 ng/g). The remaining samples with quantifiable EtG concentrations were from participants who denied use of alcohol, nicotine products, and psychoactive drugs.

### 3.5. Effect of Delayed Freezing

Samples collected on Mondays, therefore stored for up to three days before freezing, accounted for 31.5%. Compared with samples collected on other days, Monday samples exhibited slightly higher non-analyzability (28.6% vs. 21.4%) and fewer quantifiable EtG results (5.4% vs. 9.9%). However, differences were not statistically significant (Fisher’s exact test: *p* = 0.192 for non-analyzability and *p* = 0.419 for quantifiable EtG). The corresponding odds ratios were 1.47 (95% CI: 0.75–∞) and 0.51 (95% CI: 0–2.16), respectively, consistent with the observed direction of the differences. A bias due to differential degradation cannot be excluded.

## 4. Discussion

The present pilot study primarily assessed the feasibility and manageability of a protocol based on the anonymous collection of meconium-containing diapers for EtG analysis, and examined the consistency of EtG measurements in paired samples from the same newborn as an indicator of analytical reliability. As a secondary exploratory objective, the study estimated the prevalence of quantifiable EtG in neonatal meconium as an indicator of maternal alcohol exposure among participating mother–infant pairs. The results should be interpreted with caution, given several contextual, methodological, and biomarker-related limitations.

A key finding from this study is the low participation rate of about 9% among eligible mother–infant pairs, which likely introduced selection bias and limited the representativeness of the study population. Participation probably favored more health-conscious or compliant mothers, potentially leading to an underestimation of prenatal alcohol exposure and EtG positivity in the study sample.

Participation in this study was likely shaped by both methodological choices and contextual constraints. Research on alcohol use during pregnancy, particularly in settings where complete abstinence is recommended, is strongly influenced by stigma, which contributes to underreporting in questionnaires and participation bias in biomarker studies. To mitigate this, we employed an anonymous design, as prior studies have shown that anonymity improves participation and disclosure in sensitive topics. While this approach likely enhanced data validity, it prevented characterization of non-participants and assessment of differences between participants and non-participants, thereby limiting evaluation of representativeness and external validity. An anonymous opt-out approach, using parallel forms for consent and opt-out, could represent a feasible strategy to improve participation while preserving confidentiality and enabling estimation of coverage among those informed.

Contextual factors, including the sensitive nature of substance-use research, COVID-19 disruptions, postnatal wards relocation and seasonal staffing shortages may have contributed to limited recruitment.

These conditions likely disrupted communication pathways and reduced staff capacity to consistently inform mothers and collect samples, which is critical for feasibility in real-world clinical settings.

These constraints also affected the ability to rely on a priori considerations for feasibility endpoints. Consequently, the analyses were primarily descriptive and emphasized overall feasibility, aligning with the pilot nature of the study. Although an a priori power calculation for feasibility endpoints was performed, the study was ultimately restricted by recruitment challenges. Notably, this occurred during the COVID-19 pandemic, a period during which changes in health-related behaviors, including increased alcohol consumption and misuse, have been documented [17]. In addition, the anonymous study design precluded any comparison between participants and non-participants, limiting the assessment of potential selection differences and thus external validity. This limitation is particularly relevant for the secondary aim of estimating the prevalence of quantifiable EtG, whereas it has less impact on the primary feasibility objectives. Together, these factors should be considered when interpreting both recruitment patterns and the generalizability of the findings.

In Finland, studies incorporated into routine antenatal care demonstrated higher participation and offered more comprehensive estimates of PAE [18]. Within the Finnish perinatal care system, which includes structured antenatal visits and standardized neonatal care pathways, diaper-based meconium sampling could be integrated into routine postnatal care before discharge (e.g., 99.2% of births occur in public hospitals, with a mean discharge age of 3 days and a median of 2 days). The current organization of postnatal care, with short hospital stays and predominantly in-hospital births, provides a suitable framework for implementing standardized, self-collected sampling during the postnatal period. Such integration could improve participation, ensure more consistent sample collection, and reduce reliance on individual staff engagement.

Despite these challenges, the study suggests that diaper-derived meconium sampling is feasible. However, the relatively high percentage of non-analyzable samples (23%) is a significant limitation. Loss of samples due to insufficient quantity or excessive drying reduced the effective sample size and may have introduced bias if the chances of analysis were not random. This problem seems more evident than in many controlled research environments, where trained staff directly handle sample collection. These pre-analytical factors may have contributed to reduced detectability of EtG, particularly at lower concentration levels. Standardized protocols for timely sample handling are therefore essential, as delays in collection led to drying of some meconium samples in this study, compromising sample quality.

In contrast, large-scale biomarker studies conducted in Nordic and other European regions typically report higher analytical success rates due to standardized collection and immediate processing protocols [18,19,20]. These findings highlight a balance between employing non-invasive, real-world collection methods and maintaining analytical accuracy.

The observed 1.5% prevalence of EtG ≥ 30 ng/g indicates a relatively low rate of high-level exposure; however, this figure should be interpreted with caution. This estimate reflects both true exposure and methodological constraints. First, prolonged storage and delayed freezing may have caused sample degradation, reducing measurable concentrations. Temperature fluctuations during interim refrigeration could also have contributed to degradation, especially for samples stored longer, possibly affecting their integrity, particularly for samples collected on Mondays. Although no statistically significant differences were found, the observed pattern is compatible with a possible effect of pre-analytical delays, and a bias from differential degradation cannot be excluded. Second, it is well established that EtG in meconium primarily reflects alcohol exposure from mid-gestation onward, with limited sensitivity to alcohol use in early pregnancy. This is especially relevant considering Finnish research showing that even early gestational alcohol exposure, before week 7, can cause measurable biological changes, including alterations in placental DNA methylation [21]. Such early exposures would not be captured by meconium-based EtG analysis and therefore fall outside the detection scope of the present study.

The commonly used 30 ng/g cutoff is largely based on earlier studies relying on concordance with maternal self-report, which is known to underestimate alcohol use. This may have contributed to adoption of a relatively high threshold, reducing sensitivity to detect lower levels of exposure and potentially leading to underestimation of true prevalence. A large study comparing maternal self-reports with meconium biomarkers (FAEEs, EtG, and EtS) suggested that self-reported alcohol use may underestimate prenatal exposure and that combined biomarker approaches, particularly EtG and EtS, may improve detection [22]. Furthermore, EtG concentrations may be interpreted along a continuous spectrum of exposure rather than exclusively through dichotomous cutoff points. This approach would allow differentiation among varying levels of exposure, maintain quantitative information, and better support dose–response analyses in epidemiological studies. Such frameworks have the potential to enhance sensitivity, especially for low to moderate exposure levels that are below traditional thresholds. The discrepancy between self-report and EtG findings in the present study is consistent with this pattern. While very few participants reported alcohol use, a larger proportion had detectable EtG. Thus, EtG analysis in this study appears to identify exposure cases not captured by self-report. Notably, one of the highest EtG concentrations was found in a participant who did not provide information on substance use, further supporting the added value of biomarker-based approaches.

Furthermore, the study showed high intra-pair consistency (97.8%), supporting the reliability of EtG measurement even in diaper-derived meconium samples. Therefore, while pre-analytical challenges still exist, the biomarker itself is technically reliable and reproducible.

From a public health perspective, registry-based data from Nordic countries, including Finland, suggest relatively low rates of diagnosed fetal alcohol spectrum disorders (FASD), based primarily on clinically identified cases [23]. However, robust epidemiological studies indicate that the true prevalence of FASD in high-income countries with similar drinking patterns may reach 2–5% or higher [24]. This discrepancy is consistent with extensive evidence that FASD remains substantially underdiagnosed and that reliance on clinical recognition and self-report leads to significant underestimation of prenatal alcohol exposure [13].

The present study suggests that diaper-derived meconium sampling is a promising but methodologically sensitive approach. Low participation, sample loss, biomarker limits, and conservative cutoffs likely underestimate prenatal alcohol exposure. Thus, the observed prevalence should be seen as an exploratory estimate rather than a precise measure of true prenatal alcohol exposure. These influences include selection bias, pre-analytical and analytical factors affecting sample detection, and biomarker interpretation including the use of a fixed EtG cutoff.

These findings align with Finnish and international literature, which increasingly emphasizes the need for multimodal detection strategies. Future research should aim to integrate biomarker collection into routine clinical practice to improve participation, optimize sample handling to minimize degradation, including reducing delays between collection and freezing, and re-evaluate current EtG cutoff values in light of biomarker–self-report discrepancies. Moreover, combining multiple biomarkers and incorporating matrices capable of capturing early pregnancy exposure would enable a more comprehensive and sensitive assessment of prenatal alcohol exposure. Analytical advances enabling the simultaneous extraction of FAEEs, EtG, and EtS from meconium further support the feasibility of such approaches and may enhance sensitivity while extending the detectable exposure window [25]. Anonymous neonatal biomonitoring raises significant ethical considerations, particularly in balancing public health benefits with parental autonomy. It is imperative to establish transparent communication, implement appropriate consent procedures, and enforce clear governance for data use to ensure the ethical application of such methodologies.

## 5. Conclusions

In conclusion, this study showed that anonymous collection of meconium-containing diapers is feasible but operationally challenging. Ensuring sample quality and improving participation are essential for future studies. EtG remains a reliable biomarker of late-pregnancy alcohol exposure, demonstrating strong intra-pair consistency under the pre-analytical conditions of this study. These findings are consistent with previous literature reporting the relative stability of EtG in other biological matrices, such as urine, blood, and post-mortem samples [26,27], although this study did not directly assess analyte degradation. In addition, the observed low prevalence (1.5%) of quantifiable EtG in neonatal meconium provides only a preliminary indication of maternal alcohol exposure among the participating mother–infant pairs and should be interpreted with caution as an exploratory outcome.

## Figures and Tables

**Table 1:** Overview of Sample Collection and Analytical Suitability.

Sample Type	Total Samples	Not Analyzable (*N*, %)	Analyzed (*N*, %)
Single Samples	39	17 (43.6%)	22 (56.4%)
Pairs	104 (52 pairs)	12 (11.5%) (6 pairs)	92 (88.5%) (46 pairs)
One of two (from pairs)	24	12 (50.0%)	12 (50.0%)
Triplicates	3	-	3 (100%)
Unidentified	8	-	8 (100%)
Total	178	41 (23%)	137 (77.0%)

**Table 2:** Distribution of EtG levels in 137 meconium samples.

Sample Type	Analyzed	EtG < 10 ng/g	10 ≤ EtG < 30 ng/g	EtG ≥ 30 ng/g
Single Samples	22	20	2	-
Pairs	92 (46 pairs)	84 (42 pairs)	6 (3 pairs)	2 (1 pair)
One of two (from pairs)	12	11	1	-
Triplicates	3	3	-	-
Unidentified	8	8	-	-
Total	137	126 (95% CI 87–97%)	9 (95% CI 2–11%)	2 (95% CI 0–3%)

## Data Availability

The original contributions presented in this study are included in the article/Supplementary Material. Further inquiries can be directed to the corresponding author.

## Supplementary Materials

The following supporting information can be downloaded at: https://www.mdpi.com/article/10.3390/toxics14050413/s1, Supplementary Data S1. Questionnaire data, analytical results, and statistical analyses of EtG concentrations in meconium samples.
